# American Dental Society of Europe

**Published:** 1885-02

**Authors:** W. St. Geo. Elliott

**Affiliations:** London


### AMERICAN DENTAL SOCIETY OF EUROPE.

MEETING AT VEVEY, SWITZERLAND, AUG. 26, 27 AND 28, 1884.

REPORTED FOR THE INDEPENDENT PRACTITIONER BY DR. W. ST. GEO. ELLIOTT, OF LONDON.

(Concluded from page 692, Vol. V.)

A paper upon "Amalgams" was read by Dr. W. St. Geo. Elliott, (see page 62.)

*Dr. Bodecker*, (New York)—The thanks of the Society are due to Dr. Elliott for these exhaustive experiments. At a recent meeting of the New York Odontological Society, Dr. J. Smith Dodge read a paper on the tendency of amalgams to assume the spheroidal form. This tendency may account for some of the peculiar changes noticed by Dr. Elliott.

*Dr. Miller*, (Berlin)—I have seen many hundreds of copper amalgam fillings, and I have yet to find the first one which shows a tendency to draw away from the walls of the cavity or to assume a spheroidal shape. I doubt if the spheroidal tendency can be present in all amalgams.

A paper by Dr. de Trey (Vevey), entitled "Advice to Parents, etc.," was then read.

*Dr. Du Bouchet*, (Paris)—I have been in the habit of giving phosphate of lime to children to improve the character of their teeth. I would like to know the experience of others in this matter.

*Dr. Sachs*, (Breslau)—I have known parents to give lime water, and I have done so myself with very excellent results.

*Dr. Edwards*, (Madrid)—I have had some experience with the lacto-phosphate of lime. I ordered it for my wife early in gestation for the benefit of the fœtus, but I must say the results are deplorable. The child, now eight years old, has wretched teeth.

*Dr. Elliott*, (London)—My third son was brought up on condensed milk, and at four years of age his daily consumption was a tin of one pound. At that time, fearing some contraction in the throat by the exclusive use of fluid food, and because his teeth were all decaying away, I determined to force him to take more solid food, an attempt we had often made before without success. In this case it took the child four hours to swallow a small piece of bread. In the course of a week he could take solid food in small quantities, and was put upon an oatmeal diet. The decay in his teeth was at once arrested, and eleven years after he still had some of these temporary teeth. Something has been said of the injurious effects of sugar upon the teeth. It certainly neither accords with my experience nor observation. My family and myself are large consumers of sugar, and we all have remarkably good teeth.

*Dr. Miller*—Sugar when brought into contact with saliva may be converted into lactic acid, which, as you know, is very injurious to the teeth; practically, however, it is not as bad as might be supposed, since being in solution it is readily removed from the mouth by the oral secretions. Investigators have not fairly settled the question whether phosphates, etc., should be given. The effects upon bone were the same, whether more or less was taken. Only a definite quantity would be assimilated. It would be useless to give phosphates where the fault was in the assimilation; when, however, there is a simple lack of bone-forming material, then phosphates may be given with good result.

*Dr. Williams*, (Geneva)—Prof. Watt has written much on the subject, and through his influence, when associated with him in business, I gave my wife phosphates, the only result being that the teeth of the child came in rather early. Between the teeth of this child and those of another without such treatment there was no apparent difference.

*Dr. Terry*, (Milan)—My wife, during our stay at San Remo, lived mostly on fish, and the teeth and health of the child were excellent. As regards sugar, I think it injurious only when taken to excess.

*Dr. Gregory*—Mastication is my great hobby. People do not masticate enough, and suffer in consequence. I also always insist upon children sleeping with their mouths shut.

*Dr. de Trey*—I have noticed how injurious grapes are to the teeth. Many people come here for the grape cure, and I always find the teeth badly injured, mainly through excessive use of the remedy.

*Dr. Terry*—My experience with the grape cure agrees with that of Dr. de Trey; the patients overdo it. I had a case in which the enamel could almost be removed by the finger, not from the grape cure, for none were used, but from the water (medicinal) of the place. I think that the erosion we see is often the result of the tooth-brush.

*Dr. Elliott*—It is a mistake to suppose true erosion is ever caused by the tooth-brush. You know my practice is mainly among English people, and if there is one feature I have noticed more than another, it is the lack of care on the part of the patient. The people as a nation do not keep their teeth as clean as they ought to do. A large part of my time is taken up in showing them the necessity of greater care, and giving them proper directions in the use of the tooth-brush. Now I am perfectly convinced from one physical fact that erosion is not caused by the tooth-brush. It has always, or nearly so, a sharp, well defined edge. I defy any one to make a sharp edge with the brush. The edge would always be cut off; always rounded.

*Dr. Terry*—Dr. Elliott did not understand me; I only meant that injudicious use of the brush might injure the enamel; the brush should always be rotated from the gums.

*Dr. Bodecker*—As in a few minutes we are to have a clinic, and as I am to illustrate the Herbst method of operating, I will take this opportunity of explaining the process. One year ago I received a pamphlet from the Society of German Dentists at Frankfort, describing a process of burnishing foil into cavities with the engine. I saw some samples of the work, and I was amazed. I took the opportunity when in Bremen of calling on Mr. Herbst, and spent some days with him. He gave me all the information possible, and, gentlemen, I believe it is a process of value, particularly for approximal cavities. I think the gold used (that of Wolrab, of Bremen,) is too soft for either contour or crown cavities. Herbst had no experience in contouring, and asked my assistance. I prepared some cavities for him, and he filled them, and you will notice them among the specimens shown here; but either the gold is too soft, or the instrument does not fully condense it, as you can readily detect. In a competition trial with Dr. Herbst, he filled a cavity by his process in one-third the time it took me to fill a similar one with the Bonwill mallet, but my plug weighed more. The cases that we find most difficult by other modes, as the distal surfaces of molars, are the easiest by the Herbst method; but in all cases this method requires a simple cavity; if an approximal one it must be made simple by a matrix fixed solidly in position.

*Dr. Chamberlain*, (Rome)—Is the gold cohesive?

*Dr. Bodecker*—Yes, very cohesive.

The meeting then adjourned to Dr. de Trey's office, where Dr. Bodecker demonstrated on a patient the Herbst method in the distal cavity of a bicuspid.

*Dr. Bodecker*—We always use in commencing, a large point and plenty of gold; two or three large cylinders. After they have been condensed another layer is added, and so on. I do not feel sufficient confidence in myself with this new process to fill the crown, so I do that part of the operation in the usual manner.

THURSDAY MORNING.

*Dr. Rosenthal*, (Liege)—Presented through the president a new wide-mouthed bottle for holding gold foil. The peculiarity consisted in the glass stopper, which was hollow and contained chloride of calcium, or quick lime, covered with kid, the object being to absorb all moisture.

The president then called upon Dr. Elliott to read his report on Operative Dentistry.

*Dr. Elliott*—I can easily see how a paper of this kind could be made most interesting and instructive, and I think it would be a good idea if such a paper were produced every year. It should contain the gist of all the accessible professional literature of the day, both as to new books published and the magazines of all languages.

To write a paper of this kind would require much time, and while this is my idea of what the report should be, in point of fact I have not had the time to accomplish the work.

He then read some brief notes, criticising here and there, and asking for the views of those present on each subject. This led to an interesting discussion, of which we have no report.

*Dr. Elliott*—I am sorry to stop so interesting a subject, but in conclusion I am anxious to mention some experiments I have made on the subject. Believing, as I do, from much observation and many experiments, that with cohesive gold the best and most useful work we can do will be found to be the most dense in character, I have had an iron matrix made composed of two pieces, capable of being separated. The cavity was drilled out between those two parts, having a pit at the bottom to serve as a starting point. This cavity was filled in seven different ways. Each plug was then removed, weighed, and the specific gravity taken, with the results as shown below:

| | | |
|---|---|---|
| Salmon Auto-plugger. | 1 Flat Foil. | 8 thicknesses of Kearsing's No. 4 cohesive, ⅛ in. wide, annealed in advance: 9 sheets. Time 2 hours 10 minutes. Spec. Grav. 18.588+ |
| | 2 Pellets. | ¼ sheet rope cut in 8 pellets: 8¼ sheets. Time 2.24. Spec. Grav. 18.485+ |
| | 3 Crystal Gold, | Time 1.47, Spec. Grav. 17.327+ |
| | 4 Hand Pressure, | Foil same as No. 2. Time 1.30. Spec. Grav. 13.7307. |
| | 5 Bonwill Mallet, | " " " " 1.8 sh. T. 1.8. " " 15.172. |
| | 6 Herbst Method, | " " " " 1. sh. T. 1.55." " 11.560+ |
| | 7 Electric Mallet, | " " " " 1.8 sh. T. 1.30." " 15.747. |

You will see by this that in point of density the one made with the automatic and flat gold is the best, and in time the operations stand in the following order:

1, Bonwill Mallet; 2, Automatic Mallet with Crystal Gold; 3, Electric Mallet and Hand Pressure; 4, Herbst Method; 5, Automatic Mallet with Flat Gold; 6, Automatic Mallet with Pellets.

While in density they are as follows:

No. 1, 2, 3, 7, 5, 4, 6.

You know, gentlemen, our first desire is to do good work. Secondly, we wish to do it with as little discomfort to the patient as possible. We know that hand pressure is the most agreeable, and also that hand work can be made as good as any, but it is slower and much harder for the dentist, an important consideration when his physical strength is tried to the utmost by the exactions of a large practice. Often we cannot give the time that is necessary if we rely upon hand pressure (of course, I allude only to cohesive gold), consequently we must use something that will strike faster to enable us to cover the whole surface as rapidly as possible. This is, perhaps, fairly met by the several mechanical mallets now in the market—as the Bonwill, the Power, and others; perhaps most fully by the electric. Penetration does not necessarily mean efficiency. I hold in my hand a pad (2×3), composed of sheets of writing paper. On it are the footprints of the several mallets experimented with. I took a pointed plugger and the mallet I used in practice (lead, with a soft and a hard wooden face), and made a row of holes with each face, my assistant malleting with the same force as applied at the chair. I then used the automatic with the same force used in introducing the experimental fillings; then hand pressure; then the electric. The comparative penetration is shown by the number of sheets pierced, and the force applied was measured by a registering balance. In penetration the order was as follows, with the pressure in lbs., in the next column:

| | | Penetration Sheets. | Pressure in lbs. |
|---|---|---|---|
| 3¾ oz. | Mallet, soft face, wood | 3 | 2 |
| | " hard face, wood | 3 | 2 |
| 6 oz. | " lead face | 5 | 3 |
| | " leather face | 4 | 2 |
| | Hand pressure, light | 11 | 8 |
| | " " heavy | 15 | 12 |
| | Electric pressure | 4 | 2 |
| | Salmon automatic plugger | 8 | 6 |

It was a surprise to me to find that hand pressure gave the greatest penetration. My explanation is that the excellent work of the electric is not so much dependent upon the force of the blow, as upon its evenness and rapidity. This is in a measure corroborated by the results from the most regular of all blows, the automatic, necessarily regular by its construction; of course, we cannot forget at the same time that its weakness is in its slowness; evenness of condensation can only result from giving it care and time.

*The President*—As there is no time for discussion, I will call upon Dr. Elliott to read his paper on Porcelain Crowns.

*Dr. Elliott*—Mr. President and gentlemen, some five years ago I read a paper before this Society on the same subject, illustrated by some sixteen drawings, embracing all that I could find out on the subject at that date. Since then Dr. Dexter, of New York, has published in the *Dental Cosmos* very much the same thing. Dr. Butner has also published a paper. I do not claim to bring before you anything particularly new, but to call your attention to the vast amount of ingenuity that has been developed in this department, and in that way to prevent you from bringing before the profession, supposing them to be new, plans already tried and abandoned. To make the subject plain to you, and to enable us to get over a large amount of matter in a short time, I have made over sixty drawings, 2×3 feet in size, and colored so that you can see at a glance each peculiar feature. No attempt has been made to take them chronologically, the only arrangement being from the simple to the more complex.

It is impossible in this report to take up the subject in detail, and the drawings were so numerous that it is impracticable to reproduce them. The reading of the paper, or rather the necessary description and criticism attending the exhibition, lasted over an hour, and was listened to attentively by the society.

*Dr. Elliott*—I have made some experiments which may prove useful in testing the comparative stiffness of a number of metals used in making screws, pins, etc., for pivoting.

| Annealed. Lbs. | Unannealed. Lbs. | A PULL OF | |
|---|---|---|---|
| 4½ | 4½ | Bent to an angle of 45° | Platinum. |
| " | " | " " " | Phosphor Bronze. |
| " | " | " " " | Aluminum. |
| 3 | 5 | " " " | Dental Alloy. |
| 2½ | 5½ | " " " | Delta Metal. |
| 2½ | 6 | " " " | German Silver. |
| 4 | 7 | " " " | Hard Platinum. |
| " | 8½ | " " " | Steel. |

Wire No. 52 (Stubb's Wire Gauge), ¾ inch long, was used.

TABLE TO SHOW THE COMPARATIVE VALUE OF CEMENTS IN PIVOTING.

| When the pin was packed with | Lbs. |
|---|---|
| Red Gutta-Percha it pulled out at | 9 |
| Oliver's " " " | 10½ |
| Fossiline " " | 100 |
| Standard Amalgam firm at | 150 |
| Sullivan's " " | 160 |
| Rock Cement " | 170 |

Wire No. 51 (Stubb's Wire Gauge) Screw, 44 threads to the inch, was used.

The second experiment was made to show the firmness with which pins are held by different kinds of cement. A piece of iron one-half inch wide, three inches long, and one-fourth inch thick, had one-fourth inch holes drilled in a line, and each hole tapped; in the center of each, a pin with eye attached was retained by cement, and after an interval of twenty-four hours the force required to extract the pin was taken by a registering balance, and is shown in the table.

Of course we get a better result in an experiment of this kind than we can hope for in the mouth, but it shows that the ordinary practice of using oxy-chlorides and oxy-phosphates for supporting the pin is correct, making the necessary provision for the exclusion of moisture.

*Dr. Miller*—We must not accept these experiments as a positive guide as to the comparative value of different materials, as the conditions are not the same. A pin in a pivot tooth is not subjected to a pull, but to lateral motion, and the cement is liable to become disintegrated on the surface.

*Dr. Elliott*—True, but the surface should always be of something other than the white cements. Amalgam or gold should be used. This will prevent the solution, as well as disintegration. The elasticity of gutta-percha is a more theoretical than real advantage; certainly that crown which is most rigid will be most durable. Gutta-percha may last indefinitely where there is but little work for the crown to do, but where there is, the more solid it is the better.

LAST SESSION.

*Dr. Bodecker*—You know, gentlemen, that an effort has been made to raise a fund for the family of the late Dr. Webb; I am sorry to say that fund has reached but a small figure. It would be a good thing if this society were to contribute something; it would be a good example to those at home.

*Dr. Sachs*—I move that the sum of five hundred francs be sent by this society to the family of the late Dr. Webb.

*Dr. Elliott*—I second the motion.

The motion was put and carried unanimously.

The improper graduation of students by American colleges was then brought up, through the reading of a paper upon the subject, and it called out a very animated discussion. At the meeting of the society at Cologne, a committee, consisting of Dr. Williams, Dr. Cohen, and Dr. de Trey, was appointed to take the subject into consideration. But it was stated that the rascally business was still going on, and the society desired to take some measures to stop it. Europe was being flooded by unearned diplomas, coming too from legally established and hitherto respectable colleges. Our consuls had been appealed to, but they were powerless in the matter. It was proposed to go directly to the governments of Europe for help, and different gentlemen expressed a willingness to take hold of the matter. Statistics and particulars of flagrant cases had been collected, in which persons unacquainted with the English language had been graduated after a few months' attendance upon lectures which they could not understand. It was proposed that these be published, with the names of the colleges from which they obtained diplomas, but it was finally decided to await the results of a movement inaugurated in America, and to sustain any action that promised good results there. The INDEPENDENT PRACTITIONER was frequently referred to, and its action upon this subject highly commended. Berlin was designated as the next place of meeting, and the following officers were elected:

President—Prof. Dr. W. D. Miller, Berlin, Germany.
Vice-President—Dr. H. C. Edwards, Madrid, Spain.
Secretary—Dr. F. Foerster, Berlin, Germany.
Treasurer—Dr. E. de Trey, Vevey, Switzerland.

The society then adjourned.

